# Determinants of menarche

**DOI:** 10.1186/1477-7827-8-115

**Published:** 2010-09-30

**Authors:** Olga Karapanou, Anastasios Papadimitriou

**Affiliations:** 1 Third Department of Pediatrics, University of Athens School of Medicine, "Attikon" University Hospital, Haidari, Athens 12462, Greece

## Abstract

Menarche is a milestone in a woman's life as it denotes the start of reproductive capacity. Aim of this report is to review the recent developments and the current knowledge in the neuroendocrinology of pubertal onset and the factors, genetic and environmental, that influence menarcheal age. We also review the implications of early or late menarcheal age on a young woman's life.

## Background

The landmarks of the pubertal events in girls are the onset of puberty, peak height velocity (PHV) and menarche. The onset of puberty is marked by the development of breast tissue, while PHV is the highest velocity that is observed during the pubertal growth spurt.

Menarche is a rather late event in puberty and usually occurs 6 months after PHV is achieved. The age that menarche occurs varies and is dependent on the interaction between genetic and environmental factors.

In the 19th century factors that were thought to exert an influence on the physical maturation of girls were climate (particularly the mean annual temperature), ethnic origin, social status, urban or rural residence, physical activity, education, sexual stimulation, housing, inheritance, and health status [1]. Studies carried out in the 20th century documented other factors associated with the age at menarche, e.g. season and month at birth, physique, position at the sibship, family income, occupation and education of parents and family size [1].

It is considered that during the 20^th ^century the dramatic improvement of socioeconomic conditions and general health of the populations in the industrialized countries resulted in an earlier onset of puberty in children. The most reliable marker of the positive secular changes in pubertal development was the fall of the age at menarche. It has been estimated that during most of the 20^th ^century age at menarche has been falling by about 3 months per decade [2], although there are reports from industrialized countries that it has been leveling off or that it shows an upward trend [3,4].

Whatever the factors that influence pubertal maturation and age at menarche are, they interrelate and thus the onset of menarche cannot be attributed to a single factor.

Aim of this report is to review the neuroendocrinology of pubertal onset and the factors, genetic and environmental, that influence menarcheal age, and also the implications of early or late menarcheal age on a young woman's life.

We searched PubMed for relevant articles, especially published the last decade.

## Neuroendocrinology of puberty

Onset of puberty occurs after reactivation of the hypothalamic Gonadotropin Releasing Hormone (GnRH) secretory system. The GnRH secretory network initially develops and is temporarily active during fetal and neonatal life and early infancy, i.e. during the first 6 months of life, the so-called 'mini-puberty'. These early periods of GnRH activation may be important for masculinisation or feminisation of the brain [5].

**Figure 1:**
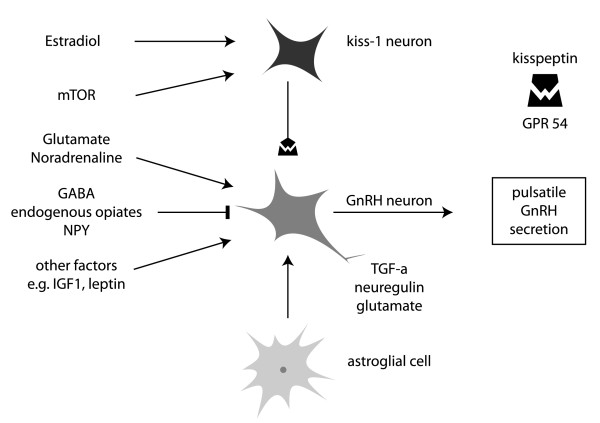
**Stimulatory (arrow) and inhibitory (blind-ended arrow) effectors of GnRH pulsatile secretion**. mTOR (mammalian target of rapamycin)

At puberty the pulsatile GnRH secretion, and the subsequent episodic pituitary gonadotropin secretion, which is necessary for normal gonadal development and function, is triggered by the activation of the GnRH pulse generator. GnRH pulse generator is comprised by scattered neurons that are distributed in the arcuate nucleus of the medial basal hypothalamus and the preoptic area in the rostral region of the hypothalamus [6]. The GnRH pulsatile secretion is dependent on the coordinated action of the scattered GnRH neurons. The latter are controlled by trans-synaptic, stimulatory and inhibitory, and glia-to-neuron inputs. Various neuropeptides and neurotransmitters have been shown to have stimulatory (e.g. gluatamate, noradrenaline) or inhibitory (e.g. γ-aminobutyric acid-GABA, endogenous opiates, NPY) [7] role in the regulation of GnRH neurons (Figure 1). The upstream genes involved in the transcriptional control of these components at the time of puberty remain to be identified. Recently, it has been established that glia-to-neuron signals include transforming growth factor-α, neuregulin and glutamergic inputs. GnRH receptors are expressed in the hypothalamic GnRH neurons suggesting that GnRH exerts an autocrine action [6,8].

Studies in human subjects with hypogonadotropic hypogonadism (HH) have disclosed a number of genes that are necessary for normal reproductive function. Among them *Kiss1 *gene, that produces Kisspeptin, and its receptor, G Protein Coupled Receptor 54 (GPR54) or Kiss1r have emerged as key players in the regulation of reproduction. Neurons that express *Kiss1 *are distributed within hypothalamus predominantly in the infundibular nucleus and scattered in the medial preoptic area [9]. Expression of *Kiss1r *transcript has been reported in human placenta, pituitary, spinal cord, and pancreas, as well as in other tissues (such as various brain regions, stomach, small intestine, etc) [10]. Kisspeptin and GPR54 are expressed in hypothalamic GnRH neurons, suggesting that both GnRHR and GPR54 may act as paracrine and/or autocrine regulators of GnRH neuronal function [11]. However, Ramaswamy et al reported that, in primates, kisspeptin-beaded axons, surprisingly, made only infrequent contacts with GnRH neurons in the mediobasal hypothalamus, whereas, kisspeptin and GnRH axons were found in extensive and intimate association in the median eminence indicating that nonsynaptic communication at the level of the median eminence is a pathway contributing to the regulation of GnRH release by kisspeptin [12]. It is evident that the hypothalamic kiss-1 system transmits metabolic information to gonadotropic axis, as expression of *kiss-1 *gene at the hypothalamus is down-regulated in conditions of negative energy balance [13]. Thus, GnRH neurons activation by kisspeptin is essential for the onset of puberty and there are data from mice that the activation of kisspeptin neurons are driven by circulating estradiol [14].

Studies of families with idiopathic HH pointed to a mutation in *GPR54 *indicating that this receptor is essential for normal GnRH secretion and pubertal development in the human [15]. Recently, an activating mutation of *GPR54 *was reported as causative of central precocious puberty in a girl [16]. It has been for more than a decade now that leptin, a hormone produced by the adipose cells, was established as exerting permissive actions on the onset of puberty by modulating GnRH neurons [17], however, the hormone is not able to initiate puberty by itself. Serum leptin levels rise in both sexes at the start of puberty. The important role of leptin in the onset of puberty is highlighted by the fact that children with Leptin gene mutations are markedly obese and delayed in puberty. Leptin is involved in the metabolic regulation of Kiss-1 as it was demonstrated that kisspeptin neurons express leptin receptors [18].

Most recently it was shown that central activation of mammalian target of rapamycin (mTOR), a serine/threonine kinase that operates as sensor of cellular energy status, stimulated LH secretion in pubertal female rats via modulation of hypothalamic KiSS-1, whereas mTOR blockade by rapamycin inhibited gonadotropic axis suggesting that central mTOR signaling has a role in the control of puberty onset and gonadotropin secretion [19].

For many years, the prepubertal quiescent period is considered to occur due to a high sensitivity of GnRH neurons (gonadostat) to the very low levels of sex steroids and to intrinsic inhibitory mechanisms within CNS that exert a blockade to the hypothalamic GnRH secretion. According to the "gonadostat " theory, the low levels of testosterone/estradiol released by the prepubertal testes/ovaries exert negative feedback effects that inhibit GnRH secretion [20]. The major inhibitory factor for GnRH release before puberty, at least in primates, appears to be **γ**-aminobutyric acid (GABA); the reduction in tonic GABA inhibition results in increase in the release of neurotransmitters, such as glutamate, which is followed by increase in pubertal GnRH release.

In view of the recent study that demonstrated that estradiol is essential for the emergence of kisspeptin expression in GnRH neurons in the prepubertal period, it was proposed that the gradual development of an estradiol-kisspeptin positive feedback relationship provides a GnRH neuron amplification mechanism that is used to facilitate the emergence of pulsatile gonadotropin secretion necessary for puberty onset.

## Assessment of menarcheal age

There are three methods for assessing age at menarche, a) the status quo, b) the recall or retrospective, and c) the prospective methods [21]. In the status quo method data regarding menarcheal age can be obtained by asking a girl (or her parent) of her "current status", i.e. whether she has had her first menses by the time of assessment, and her birth date. In the status quo method the sample must be large, representative of the population, and in the developed countries the age range should be from 8 to 16 years old.

In the recall method menarcheal data are obtained by asking post-menarcheal females (or their mothers) to recall their age at first menses. The recall method may be less valid and its accuracy is decreased with greater time elapsed between menarche and asking for the date, because it is fraud with poor memory. Furthermore, all girls included must be at an age that they normally should have already started menstruating.

The prospective method is more accurate, however such studies are not easy to perform as they should be longitudinal having premenarcheal girls followed regularly, ideally every 3 months, and asked at each visit whether they have begun to menstruate. Therefore, most studies on menarcheal age have employed the status quo or the recall methods.

There are numerous studies (Table 1) examining the secular trend of age at menarche in various populations. In general, there is a continuous trend for earlier ages at menarche for the most part of the 20^th ^century, although this trend tends to slow down or stabilize. In the United States, the mean age at menarche was more than 14 years prior to 1900 [22] and it decreased to 12.43 years in a study conducted between 1988 and 1994 [23], although there were significant racial differences in maturational timing. In the Fels Longitudinal Study girls born in 1980 had menarche at 12.34 years of age suggesting a further decrease in menarcheal age [24]. Euling et al assessed the data for evidence of a still existing secular trend in puberty timing for data collected from 1940 to 1994 in US girls [25]. These data suggest a trend towards an earlier onset of breast development and menarche but not for other female pubertal markers.

**Table 1:** Age at menarche in various countries around the world^1^

Country, Date: Age	
Italy 1995:12.0	India 1998: 12.1
Greece 1999: 12.3	Hong Kong 1997:12.4
Thailand 1997: 12.5	USA/NHANES^2 ^2001: 12.5
France 2006: 12.6	Japan 1992: 12.6
Spain 2002:12.6	Venezuela 2000: 12.6
Denmark 1998: 13.0	Finland 1993: 13.0
UK 1993:13.0	Belgium 1985:13.1
Cameroon 1999: 13.2	Netherlands 2000: 13.2
South Africa 1990: 13.2	Sweden 1996: 13.2
Switzerland 1983: 13.4	Germany 1996: 13.5

^1^Adapted from Ref [38]

^2^National Health and Nutrition Examination Survey

## Maturational tempo differences and age at menarche

A question that arises is what is the association between timing of onset of puberty and menarche, in other words will girls with early puberty invariably have menarche at an early age and vice versa? Data gathered from women born between 1977 and 1979, showed only a moderate correlation between menarche and onset of puberty (.37-.38), whereas in earlier studies conducted on women born between 1920 and the 1960s much higher correlations (.64-.86) were reported [26]. This may be explained by a change in the maturational tempo in girls born after the 1970s. Indeed, studies carried out during the last 10 years in Spain, the USA and Greece [27-29] showed that early maturing girls present a compensatory delay in pubertal progression that could explain the moderate correlation between the age at menarche and at pubertal onset.

These studies have shown that early pubertal maturation in girls is associated with a greater pubertal height gain and a longer period of pubertal growth, which is due to a longer pubertal growth up to the stage of peak height velocity, whereas after that stage there is no significant difference to the duration of pubertal growth irrespective of the timing of the onset of puberty [29].

## Genetic determinants of menarche

Menarcheal age is influenced by heredity but the specific genetic determinants are largely unknown. Evidence for hereditary influences on the age at menarche comes from studies that show a trend for maternal age at menarche to predict daughter's age at menarche [30]. In fact, approximately half of the phenotypic variation among girls from developed countries in the timing of menarche is due to genetic factors [31].

In search for specific genes determining the timing of menarche Stavrou et al [32] evaluated whether the XbaI and PvuII polymorphisms of estrogen receptor a (*ERa*) gene are associated with the age at menarche. After adjusting for body mass index this cohort demonstrated that the difference between XX homozygotes and other subjects was 0.57 years (p = 0.021). The difference between PP homozygotes and other subjects was not significant (0.26 years, p = 0.26), while the difference between PX homozygotes and other subjects was 0.67 years (p- = 0.008), suggesting that XbaI XX homozygotes or, in more general terms, subjects homozygotes for the PX haplotype seem to have a modest delay in the age of menarche. Regardless of the exact mechanism, if *ERa *gene polymorphisms can alter the estrogenic biological activity at the cellular level, this may influence the maturation of the hypothalamic-pituitary-gonadal axis, which determines the onset of menarche [29]. On the other hand, investigation for *GnRH *receptor mutations revealed that genetic variation in *GnRH1 *and *GnRHR *is not likely to be a substantial modulator of pubertal timing in the general population [33].

Recently, a genome-wide association (GWA) study was conducted aiming to identify common variants associated with the timing of puberty [34]. For age at menarche only one single nucleotide polymorphism reached genome-wide statistical significance, rs314276 in intron 2 of *LIN28B *on chromosome 6. Each major allele was associated with 0.12 years earlier menarche. This allele was also associated in girls with earlier breast development, in boys with earlier voice breaking and a faster tempo of growth and shorter adult height in both sexes.

## Ethnic/racial differences in the timing of puberty and menarche

Several studies performed, especially in the USA, have shown ethnic-racial differences in pubertal maturation and menarche. Black girls were younger than white girls at the same stage of breast development, pubic hair development and menarche.

The Bogalusa Heart Study included data from 7 cross-sectional examinations of school-aged children, which were used for both cross-sectional and longitudinal analyses [35]. According to the study, black girls experienced menarche, on average, 3 months earlier than did white girls (12.3 vs 12.6 years) and during a 20 year study period, the median menarcheal age decreased by approximately 9.5 months among black girls. Secular trend among white girls was smaller and less consistent, since a 2 month decrease was recorded.

Anthropometric dimension differences between black and white girls, such as weight, height, and skinfold thickness, could be a confounding factor; however, controlling for height and either BMI or weight, the rate of early menarche remained significantly higher among black girls, suggesting that race is an independent factor of pubertal/menarcheal timing. The racial difference in pubertal maturation may reflect genetic factors. Black girls present higher insulin response to a glucose challenge, and subsequently increased levels of free IGF1, which is associated with skeletal and sexual maturation compared to white girls [36].

Moreover, another US study performed using data from the Third National Health and Nutrition Examination Survey, demonstrated that the mean age at onset of pubic hair, breast development and menarche was 9.5, 9.5, and 12.1 years for black girls; 10.3, 9.8, and 12.2 for Mexican American girls; and 10.5, 10.3 and 12.7 for white girls. The racial/ethnic differences remained even after adjustment for current body mass index and several social and economic variables [37].

Girls in South Europe experience menarche earlier than girls in North Europe. Mean menarcheal age in France and other Mediterranean countries is lower than in other Western European countries [38], which points to a geographical difference that reflects both genetic or ethnic and environmental factors. Geographical differences might involve altitude, temperature, humidity and lighting. Of special interest is that lighting signals on hypothalamus- pituitary- gonadal axis are supposed to be mediated through melanotonin circuit. According to several studies, menarche is more frequent in winter than in summer, which points to an inhibitory effect of photostimulation.

Age at menarche in Asian is similar to Mediterranean girls; mean menarcheal age in Hong Kong [39] and Japan [40] is 12.38 and 12.2 years, respectively, and in Greece [41] or Spain [42] 12.27 and 12.34, respectively.

## Menarcheal age related to body fat, nutrition and physical activity

Body size parameters, such as weight or BMI and height are strongly correlated with the age at menarche. Frisch and Revelle proposed a critical body weight and weight gain for the onset of menarche [43,44]. Higher subcutaneous fat levels and BMI at prepubertal ages (5-9 years) are associated with increased likelihood of early (<11 years) menarche [35]. Age at menarche is negatively related to hip and thigh circumference and positively related to waist circumference, status and biiliac breadth [45]. Blood leptin levels were also much more strongly related to gluteofemoral than upper body fat, suggesting that leptin may convey information about fat distribution to the hypothalamus during puberty [45]. The association of birth weight with pubertal development has not been yet well documented [46,47], although there are data that in small for gestational age girls the age at both pubertal onset and menarche are advanced by about 5-10 months, whereas in boys pubertal onset usually occurs at an appropriate age [48]. On the other hand, Ellison related menarcheal age to height rather than weight, suggesting that skeletal maturation is more important than body fat accumulation for menarche [49], a finding that was also supported by a survey performed among schoolgirls in Taiwan [50].

Other studies have shown that rapid early infancy weight gain from birth to age 2 months, and also from 2 to 9 months, predicted subsequent greater adiposity, assessed by DXA at age 10 yr, and earlier age at menarche in girls. This association was not existent when weight gain occurred later in infancy, i.e. between 9 and 19 months of age [51].

Regarding nutritional habits, increased energy-adjusted intake was associated with early menarche [52]. The quality of food intake also influences puberty. Berkey et al [53] demonstrated that high animal versus vegetable protein ratio at the ages of 3-5 years is associated with early menarche, after controlling for BMI.

A cross sectional study performed in a group of Colombian university women demonstrated that age at menarche was positively associated with the practice of at least two hours daily of physical activity [54]. Menarche, on average, occurs later in athletes, including ballet dancers, than in the general population, with the exception of swimmers, suggesting that intense exercise delays puberty [55]. The most probable explanation for no delay in menarcheal age of swimmers is that the normal body fat composition of swimmers balances the negative hypothalamic effect on GnRH pulsatile exerted by intensive exercise.

## Environmental influences on pubertal/menarcheal timing alteration

Socioeconomic factors or life setting, such as urban/rural residence, family size, family income, level of parental education, may also influence pubertal development. Girls from families with a high socioeconomic status experience menarche at an earlier age than girls from families with lower socioeconomic status [56]. Furthermore, higher parental education has been associated with earlier timing of puberty [56]. Absence of a biological father, the presence of half- and step-brothers is associated with early menarche, whereas the presence of sisters, especially older ones, in the household while growing up, was associated with delayed menarche [57]. The prevalence of early menarche is even higher when stepfather presence is combined with a stressful family environment and with maternal mood disorders [57]. Girls raised in urban environments have earlier menarcheal ages than those raised in rural environments [58].

Third World girls adopted in Western European countries present the pattern of early menarche, which indicates the role of transition from an underprivileged to a privileged environment as determinant of menarche [59]. It has been suggested that the premature sexual maturation may be triggered by the catch-up growth that these children present [60]. Other stresses like acute/chronic illnesses [61] or war conditions [62] suppress the hypothalamic- pituitary-gonadal axis and delay pubertal onset.

Various studies focus on the role of environmental chemicals, called endocrine-disruptor chemicals (EDC), on puberty timing alteration. EDC are used as industrial solvents/lubricants [polychlorinated biphenyls (PCBs), polybrominated biphenyls (PBBs), dioxins], plastics [bisphenol A (BPA)], plasticizers (phthalates), pesticides [methoxychlor, chlorpyrifos, dichlorodiphenyltrichloroethane (DDT)], fungicides (vinclozolin), and pharmaceutical agents [diethylstilbestrol (DES)] [63]. Endocrine disruptors may have structural similarity with estrogen, thus their action is performed through estrogen receptor, whereas others, e.g. vinclozolin, interact with the androgen receptor, while other EDC, e.g. fadrozole and ketoconazole, inhibit aromatase or steroidogenesis, respectively. Furthermore, EDC may affect puberty via CNS regulation. For example, atrazine delays puberty in both males and females, reducing circulating LH and prolactin [64]. Phthalates and polychlorinated biphenyls have been associated with earlier breast development and menarche, respectively [65]. EDC may also influence the endocrine system in a harmful manner, inducing human disorders of sex differentiation [66] and hormone- dependent cancers [67].

## Health implications of early or late menarche

Early puberty is associated with increased body mass index, insulin resistance, total number of metabolic syndrome components and hence increased cardiovascular risk [68]. Moreover girls with early menarche exhibit elevated blood pressure and glucose intolerance compared with later maturing girls, independent of body composition [69]. A recent large population-based Caucasian cohort study also confirmed these observations, but also correlated in a non-linear significant manner the earlier age at menarche (<12 yrs) with higher incident cardiovascular disease, incident coronary heart disease, all-cause mortality and cancer mortality after adjustment for age, physical activity, smoking, alcohol, educational level, occupational social class, oral contraceptive use, hormone replacement therapy, parity, BMI and waist circumference. Most of the subjects had a positive family history of heart attack, which indicates that this association may be partly mediated by increased adiposity but genetic mechanisms may contribute as well [70].

It has also been reported that menarcheal age influences bone mineral density and consequently the incidence of lumbar spine and hip fractures. Girls menstruating earlier have slightly higher bone mineral density of the lumbar spine and femoral neck in older age after excluding subjects who took hormone replacement therapy and adjusting for BMI [71]. This might be apparently explained due to longer lifetime exposure to the protective effects of endogenous estrogens at the period of acquisition of peak bone mineral density. On the other hand, late menarche is associated with osteoporosis, since studies including postmenopausal women suggest that those having later menstruated have lower mineral density at forearm, spine and proximal femur and increased risk of fractures as well [72,73]. Moreover, apart from older women, onset of menstruation is a determinant factor of peak bone mineral density in young women. According to a Japanese cohort study, late menarche (>14 yrs) is associated with approximately 2- fold increased risk of low areal bone mineral density at the hip in women aged under 40 years [74]. Age at menarche correlates not only with bone mineral density but also with bone microstructure, since young healthy women with late menarche display lower total volumetric bone mineral density, cortical volumetric bone mineral density, and cortical thickness at forearm, a finding compatible with less endocortical accrual [75]. Finally, recent data suggest that in girls experiencing menarche later, the deficit of areal bone mineral density is generated before the onset of pubertal maturation with very mild increment during the whole period of pubertal maturation. This observation indicates that estrogen exposure is not the only key factor responsible for the influence of menarcheal age on peak bone mineral density and that other genetic determinants could be involved [76].

Multiple studies confirm that early menarche is a risk marker for breast cancer [77]. This predisposition is enhanced by the observation that earlier onset of menarche is accompanied by abdominal- type obesity and thus higher circulating levels of insulin, testosterone and insulin- like growth factor 1, which in turn act as growth factors for mammary tissue proliferation and are likely to promote mammary gland carcinogenesis [78].

Early menarche leads to earlier sexual intercourses and is a risk factor for adolescent depression [79]. Girls associate a variety of negative physical and psychological changes with menstruation, reflecting both misconception and ignorance [80] and the fear of being different from peers as well [81]. Girls' attitudes and expectations about menstruation are negatively biased and have been found to contribute to self- objectification, body shame, and lack of agency in sexual decision making [82]. Early maturing adolescents with problematic peer relations experience elevated social anxiety symptoms [83]. A recent research combined accelerated sexual maturation with negative experiences with the opposite sex, indicating that the earlier the menarche, the larger the estimated egocentric distance of virtual male voices and the more negative the evaluations of male faces [84]. Moreover, if early maturation is combined with social factors, such as an underprivileged neighbourhood, susceptibility to a violent behaviour enhances [85]. On the other hand, girls with constitutional delay in puberty and onset of menstruation feel that this delay has an impact on school, work or social status and would prefer to accelerate their growth spurt by treatment [86].

A summary of the health implications of early or late menarche are shown on Table 2.

**Table 2:** Health implications of early or late menarcheal age

Early menarche	Late menarche
Abdominal type obesity	Osteoporosis
Insulin resistance	Adolescent depression
Glucose intolerance	Social anxiety symptoms
Cardiovascular risk	
Coronary heart disease	
Increased bone mineral density	
Increased cancer mortality	

## Conclusions

The improvement of socioeconomic conditions that took place in the 20^th ^century resulted in an earlier onset of puberty in children, indicated by the fall of the age at menarche. However, there are reports from industrialized countries that this trend has been leveling off.

Onset of puberty is controlled by a complex neuronal network, *Kiss1 *gene, that produces Kisspeptin, and its receptor, G Protein Coupled Receptor 54 (GPR54) having a key role in pubertal onset and reproduction. Recently, a single nucleotide polymorphism of *LIN28B *on chromosome 6 was associated with earlier menarche.

Besides genetics, menarche is also influenced by socioeconomic and environmental factors. Race, BMI, geography, nutritional habits, exercise all have been shown to influence menarcheal age; furthermore, Third World girls adopted in developed countries present early menarche. Moreover, endocrine-disruptor chemicals result on puberty timing alteration.

Menarcheal age has important health implications, as early menarche is associated with more cardiovascular incidents and higher all cause, including cancer, especially of the breast, mortality. Late menarche is associated with osteoporosis and increased fracture risk. Moreover, early menarche has been related to anxiety symptoms, depression, premature intercourse and violent behavior.

More studies are needed in order to predict which girls may develop metabolic or psychological disturbances due to early menarche and whether they can be benefited by medical manipulation of the pubertal events.

## Competing interests

The authors declare that they have no competing interests.

## Authors' contributions

AP conceived of the report, helped to draft the manuscript and in figure and tables' design. OK carried out literature research and drafted the manuscript and the tables as well. Both authors read and approved the final manuscript.
